# Supplementary material for: Cost-utility analysis of botulinum toxin type A versus oral drug treatment in patients with severe blepharospasm in Thailand
Source: PLoS One. 2025 Apr 21;20(4):e0319926. doi: 10.1371/journal.pone.0319926 (PMC12011269; doi:10.1371/journal.pone.0319926)
Supplement: S1 Table — (DOCX) [file pone.0319926.s001.docx]

Table S1: The demographic data of reference source

| **General** | **N=159** |
| --- | --- |
| Age (years) (Mean, SD) | 61.40 (10.09) |
| Sex (Female) | 88.05% |
| Presence of Underlying diseases | 71.70% |
| DM | 15.09% |
| HT | 36.48% |
| DLP | 32.70% |
| Old CVA | 0.63% |
| Thyroid diseases | 1.89% |
| CAD | 1.89% |
| CKD | 1.26% |
| Other | 26.42% |
| Number of previous injections | |
| 0 | 11.95% |
| 1-5 | 21.38% |
| 6-10 | 18.24% |
| 11-20 | 28.30% |
| >20 | 20.13% |
| Jankovic Rating Scale; JRS (Mean, SD) | |
| Frequency grading | 3.40 (0.49) |
| Severity grading | 3.41 (0.49) |
| Total grading | 6.81 (0.87) |
| Presence of BEB-related accident | 12.58% |
| Oral medication for BEB | 18.87% |
| New case | 10.06% |
| Employment | 60.38% |
