# Supplementary material for: Cost-utility analysis of botulinum toxin type A versus oral drug treatment in patients with severe blepharospasm in Thailand
Source: PLoS One. 2025 Apr 21;20(4):e0319926. doi: 10.1371/journal.pone.0319926 (PMC12011269; doi:10.1371/journal.pone.0319926)
Supplement: Attachment — Submitted filename: Reviewer comments.docx [file pone.0319926.s002.docx]

Thank you for the opportunity to review this article. The study is well-designed and written. However, some comments should be considered by the authors.

1. The authors should add more information in the introduction about the national burden of Blepharospasm in Thailand. This will justify the study question
2. In the abstract, what does the author mean by hybrid model? This is not well-known in pharmacoeconomic studies. If they mean decision tree followed by Markov then please mention it.
3. Is there any population receive supportive care? It is important to mention what is the standard practice in Thailand.
4. How you confirmed that the study sample is generalizable to Thailand population? Please add a table for patient demographics.
